# Influence of Work Values on the Prescribing Behavioral Intentions Regarding Antibiotic Use Among Primary Physicians in Hubei, China

**DOI:** 10.3389/fpubh.2022.830181

**Published:** 2022-05-13

**Authors:** Junyu Lu, Chenxi Liu, Dan Wang, Xinping Zhang

**Affiliations:** ^1^School of Medicine and Health Management, Tongji Medical College, Huazhong University of Science and Technology, Wuhan, China; ^2^School of Management, Hubei University of Chinese Medicine, Wuhan, China

**Keywords:** antibiotic prescribing, work values, behavioral intentions, primary physicians, China

## Abstract

**Objective:**

Primary physicians have been an important cause of global antibiotic resistance. The aim of this study is to identify the influence of primary physicians' work values on prescribing behavioral intentions regarding antibiotic use (behavioral intentions).

**Methods:**

A total of 656 primary physicians' work values and behavioral intentions were collected by a stratified cluster sampling from 67 primary care facilities in Hubei Province of China. Work values included 5 dimensions, namely intrinsic values, extrinsic values, reward values, social values and altruistic values. Behavioral intentions included 2 dimensions of the intentions to reduce antibiotic prescriptions and the intentions to prescribe antibiotics. A Likert five-point scale was used and higher scores meant greater intentions to prescribe antibiotics. A hierarchical multiple regression analysis was employed to model the influence of work values on behavioral intentions.

**Results:**

Primary physicians' behavioral intention was 2.01 averagely. Intrinsic values negatively influenced overall intentions to prescribe more antibiotics (β = −0.098, *P* = 0.010). Whereas lower social values perception (β = 0.248, *P* < 0.001), less pursuit of reward values (β = 0.194, *P* < 0.001), and less emphasis on altruistic values (β = 0.180, *P* < 0.001) positively influenced lower overall intentions to prescribe antibiotic prescriptions. Besides, extrinsic values were not found influencing the behavioral intentions (β = 0.001, *P* = 0.961).

**Conclusions:**

Primary physicians' work values influenced their behavioral intentions regarding antibiotic use. Training and education of work values may be an entry point for intervention on improving antibiotic prescribing.

## Introduction

The emergence and increase of antibiotic resistance has become a significant public health threat, with a series of serious consequences, for example, it was estimated that 10 million people worldwide would die of antibiotic resistant infections in 2050, and infection caused by antibiotic resistance resulted in prolonged hospital stays, increased mortality and high health care costs (1–7). One of the important causes of antibiotic resistance is physicians' inappropriate prescription, which has been a severe problem in primary care facilities (8, 9). For instance, the proportion of outpatients encountered antibiotic prescriptions reached 53.4% in primary care facilities in China, seriously exceeding the proportion recommended by WHO (30%) (3). Therefore, many researches have been done to find the reasons and strategies for inappropriate use of antibiotics in primary care facilities (10, 11).

Recently, researchers have attempted to identify work values as important determinants of individual work-related behaviors (12, 13). Although physicians' pivotal role has been explored for in-depth understanding why they prescribe antibiotics inappropriately from the perspective of values mapping such as working environment, patient requirements and reward incentives, studies that systematically measure physicians' work values regarding antibiotic prescribing behaviors are still insufficient (11, 14–16).

As a vocational concept, work values emerge from the projection of general values onto the work setting (17), and show a perception of preference for certain aspects of individuals' work behaviors. Work values play important roles in work-related motivations and the establishment of goals that individuals intend to achieve, which lead an individual toward a particular role (13, 18). Most researchers have explored three or four types of work values: (1) intrinsic or cognitive values, pertaining to intrinsic personal orientation that affects work, such as independence and use of abilities; (2) extrinsic or instrumental values, pertaining to material aspects of work, such as work conditions; (3) social or affective values, pertaining to interaction with work-related people, such as co-workers and patients; (4) reward or prestige values, pertaining to a sense of personal superiority gained from work, such as advancement, achievement, and a prestigious, highly valued work (18–21). Besides, altruistic values related to the desire to make contributions to society and help others, were incorporated into work values, with the significance that workers would pay more attention to helping others especially in medical field (18, 21–23).

The research of influence of work values on behavior were mainly limited in the fields of post-retirement work intentions, entrepreneurial intentions, profession choices and turnover intentions (24–29). For example, workers expressed more post-retirement work intentions if they perceived the value of work to fit their personal value orientation (25). Entrepreneurial intentions were influenced by some dimensions of work values (24, 26). Besides, nurses' profession choices and turnover intentions were also found to be associated with work values (27–29). However, we did not find studies on the influence of work values on primary physicians' medical behaviors, especially the behaviors regarding antibiotic use.

The objectives of this study were to explore the influence of primary physicians' work values on their behavioral intentions regarding antibiotic use, in order to make contribution to the improvement on public health and antibiotic stewardship.

## Methods

### Data Collection

A stratified cluster sampling method was used to collect data. The clusters were primary care facilities covering urban community health centers (UCHCs) and rural township health centers (RTHCs)in Hubei Province, which is geographically divided into western, central, and eastern regions. Based on the strategies developed by Dhand and Khatkar (the expected deviation < 6, precision = 1, level of confidence = 95%, correlation coefficient within the group < 0.02 and cluster size = 10) (30), we estimated that each region required at least 17 clusters. In each region, one urban city and two rural counties were randomly selected. Eight primary care facilities (UCHCs or RTHCs) were then randomly selected in each selected city or county. To ensure sample size, more than 70 primary physicians were interviewed in each city/county. If the number of respondents in the selected city/county was < 70, an additional primary care facility was added to the sample. This sampling method was also used in other studies by our team (31, 32). Finally, a total of 67 primary care facilities, including 19 UCHCs and 48 RTHCs were enrolled in this study.

A structured self-administered questionnaire was applied to a cross-sectional survey from 23 April 2018 to 6 June 2018. Based on the composition of departments in primary care facilities in China, all primary physicians on duty with antibiotic prescribing authority from general practice, internal medicine, pediatrics, surgery, gynecology and Chinese medicine departments were invited to participate in this study. The investigators were trained strictly for the survey quality and distributed 712 questionnaires to the primary physicians, who were asked to submit informed consents and then complete the questionnaires independently. The incomplete questionnaires were returned to the physicians for recompletion. Eventually, a valid sample size was 656.

### The Measurement

#### Work Values

Work values were measured from the following 5 dimensions, with a total of 16 items, based on the work value scales established (18–21, 33). Intrinsic values covered 2 items as the independence and use of abilities in work, in which higher score, more independently and freely that primary physicians could prescribe antibiotics and use of their professional abilities in work (19, 20). Extrinsic values covered 3 items as the work condition on bacterial identifications, drug sensitivity tests and general work condition, in which higher score, worse work condition that primary physicians perceived (19, 21). Reward values covered 3 items as the correlation between career promotion and prescription behavior, continuous learning and gain of prestige, in which higher score, higher rewards that primary physicians pursued (18, 21). Social values covered 4 items as the social pressures, consisting of recognition, trust, requests from patients and interaction with co-workers, in which higher score, more social pressures primary physicians perceived (18, 20, 21). Altruistic values covered 4 items as the contribution to patients, help patients appropriately, no harm to patients and do a responsible work, in which higher score, more likely physicians perceive altruistic from their prescribing behaviors (18, 19). For each item, primary physicians were asked to rate the agreement on a Likert five-point scale from 1 (strongly disagree) to 5 (strongly agree).

A pilot survey was conducted to assess the reliability and validity of the questionnaire on work values. The Kaiser–Meyer–Olkin value = 0.723 (KMO > 0.70, acceptable), and the Bartlett test *P* < 0.001 (*P* < 0.05, acceptable) (34), verified the feasibility of the exploratory factor analysis. The reliability of responses was further assessed using Cronbach's alpha. Coefficients of 0.62 or better were obtained in each sector for each of the 5 dimensions of items. Spearman rank correlation value was to evaluate the correlation between items and their corresponding dimensions, ranged from 0.55 to 0.91, with *P* < 0.001 (< 0.05, acceptable). Regarding to the confirmatory factor analysis, a good fitness of the questionnaire was found: Tucker-Lewis index = 0.96 (TLI > 0.90, acceptable), comparative fit index = 0.96 (CFI > 0.90, acceptable) and the root mean square error of approximation = 0.04 (RMSEA < 0.08, acceptable) (35).

#### Behavioral Intentions

The Theory of Planned Behavior proposes a model about how human action is guided. It predicts the occurrence of a specific behavior and regards intention as the precursor of action (36). Although there is a certain deviation between the behavioral intention and the actual behavior, intention can be used as an approximate measure of behavior when the measure of actual behavior is not readily available (35, 36). In this study, behavioral intentions represented the degree that primary physicians felt like to prescribe antibiotics and was computed by the averaging of next 4 items. The first three items reflected the behavioral intentions of primary physicians toward “expect”, “want” and “intend” to reduce antibiotic prescriptions. The rating was performed on a five-point Likert scale from strongly agree (score = 1) to strongly disagree (score = 5). The last item was the intention to prescribe antibiotics to patients, which was measured by the number of patients (from 0 to 10) that would be prescribed with antibiotics. And the behavioral intentions were classified into 5 categories: patients ≤ 2 were defined as 1 point; others were equally divided into 4 groups, with a score of 2 ~ 5 points according to the rational use rate (< 30%) of antibiotic prescriptions in primary care facilities recommended by WHO (37). The lower the scores, the better behavioral intentions.

A pilot survey was conducted to assess the reliability and validity of the questionnaire on behavioral intentions. The Cronbach's alpha coefficients was 0.90, with good internal consistency.

### Statistical Analysis

The mean and standard deviation of each item were to describe work values and behavioral intentions. Hierarchical multiple regression analysis was conducted to determine the influences of work values on the intentions to reduce antibiotic prescriptions (Model 1), on the intentions to prescribe antibiotics to patients (Model 2) and on the overall behavioral intentions regarding antibiotic use (Model 3). The three models were designed respectively as 2 kinds of models. In the first kind of models as M11, M21, and M31, gender, age, education, working experience and department were entered as controlled variables; in the second kind of models as M12, M22, and M32, both the controlled variables and work value dimensions' measures were entered into the models. The standardized regression coefficient (β) of each variable and the corresponding *P*-values were calculated for multivariate analyses. The proportion of variance in behavioral intentions that could be explained by work values was assessed by the coefficient of determination (R^2^) (38). Significance was considered at 2-sided P < 0.05.

The statistical analysis was performed using SPSS (version 24.0) and Amos (version 24.0).

## Results

### Characteristics of Primary Physicians

Characteristics of primary physicians are presented in Table 1. The average age was 43.26 years (SD = 10.41), and males occupied the majority (69.36%). The years of working experience on average was 16.66 (SD = 11.06). Most of the physicians came from RTHCs (77.90%).

**Table 1:** Characteristics of primary physicians.

**Characteristics**	**Sampled visits**	***N* (%)**	**Mean±SD^a^**
Age (years)			43.26 ± 10.41
Gender			
Male	455	69.36	
Female	201	30.64	
Facility			
Urban community health center	145	22.10	
Rural township health center	511	77.90	
Education			
Associate degree and below	404	61.59	
Bachelor degree	250	38.11	
Master degree	2	0.30	
Department			
General practitioner	264	40.24	
Internist/pediatrician	160	24.39	
Surgeon	92	14.02	
Gynecologist	90	13.72	
Chinese medical practitioner	50	7.62	
Professional title			
Junior doctor	341	51.98	
Attending doctor	250	38.11	
Associate senior doctor	61	9.30	
Senior doctor	4	0.61	
Working experience (years)			16.66 ± 11.06

a *SD, Standard deviation*.

### Work Values and Behavioral Intentions

Table 2 presents the levels of 5 dimensions of work values and 2 dimensions of behavioral intentions. The primary physicians displayed variations in work values. Extrinsic values (Mean=4.41, SD=0.83) presented the highest mean scores across all dimensions. Intrinsic values (Mean=3.46, SD=0.78), altruistic values (Mean = 3.22, SD = 0.81) and reward values (Mean = 3.11, SD = 0.77) presented slightly high scores. However, the scores of social values were the lowest (Mean = 2.08, SD = 0.59).

**Table 2:** Work values regarding antibiotic use and behavioral intentions.

**Work values/Behavioral intentions**	**Mean**	**SD^a^**	**Cronbach's alpha**	**r^b^**	**P^c^**
***Work values***	3.26	0.35			
**Intrinsic values**	3.46	0.78	0.60		
Independence in prescribing antibiotics	3.24	1.06		0.91	<0.001
Being able to use of abilities	3.69	0.79		0.75	<0.001
**Extrinsic values**	4.41	0.83	0.68		
Hard to receive bacterial identifications	4.59	1.09		0.60	<0.001
Hard to receive drug sensitivity tests	4.45	1.29		0.67	<0.001
Bad work condition in primary care facilities	4.19	0.76		0.80	<0.001
**Reward values**	3.11	0.77	0.58		
Low correlation between career promotion and prescription behavior	2.43	0.94		0.60	<0.001
Few opportunities for continuous learning	2.83	0.98		0.55	<0.001
A prestigious, highly valued work	4.09	1.64		0.70	<0.001
**Social values**	2.08	0.59	0.67		
Work for recognition	1.62	0.81		0.70	<0.001
Work for trust	1.38	0.70		0.71	<0.001
Work at requests of patients	2.70	1.07		0.63	<0.001
Work after interacting with co-workers	2.63	0.89		0.62	<0.001
**Altruistic values**	3.22	0.81	0.92		
Contribution to patients	3.37	0.86		0.86	<0.001
Help patients appropriately	3.24	0.92		0.89	<0.001
No harm to patients	3.20	0.98		0.86	<0.001
A responsible work	3.06	0.90		0.90	<0.001
***Behavioral intentions***	2.01	0.61			
**Intentions to reduce antibiotic prescriptions**	1.70	0.54	0.90		
I expect to reduce antibiotic prescriptions to patients	1.66	0.58		0.91	<0.001
I want to reduce antibiotic prescriptions to patients	1.65	0.57		0.90	<0.001
I intend to reduce antibiotic prescriptions to patients	1.80	0.61		0.92	<0.001
**Intentions to prescribe antibiotics to patients^d^**	2.32	1.02	N/A		

a *SD, Standard deviation;*

b *r, Spearman rank correlation value;*

c *P, P < 0.05 indicates statistical significance;*

d *Intentions to prescribe antibiotics to patients, The average prescription was 3.98, with an average score of 2.32 in 5 categories*.

The average score of behavioral intentions was 2.01 (SD = 0.61). Primary physicians intended to prescribe antibiotics to nearly 40% of patients (3.98 patients, Mean = 2.32, SD = 1.02), and they showed intentions to reduce antibiotic prescriptions (Mean = 1.70, SD = 0.54).

### Influence of Work Values on Behavioral Intentions

The influence of work values on primary physicians' behavioral intentions regarding antibiotic use were better explained by M12, M22, and M32, because the R^2^ values in the M12, M22, and M32 were considerably higher than those in M11, M21, and M31.

According to the hierarchical multiple regression analysis, four dimensions of work values were statistically associated with behavioral intentions. Intrinsic values negatively influenced overall intentions to prescribe more antibiotics (β = −0.098, *P* = 0.010), while social values perception (β = 0.248, *P* < 0.001), reward values (β = 0.194, *P* < 0.001), and altruistic values (β = 0.180, *P* < 0.001) positively influenced overall intentions to prescribe more antibiotics. Besides, extrinsic values were not found to influence the behavioral intentions (β = 0.001, *P* = 0.961). The detailed results are summarized in Table 3.

**Table 3:** Influencing factors of work values toward behavioral intentions.

**variables**	**Model 1**		**Model 2**		**Model 3**	
	**Reduce antibiotic prescriptions**		**Prescribe antibiotics to patients**		**Overall behavioral intentions**	
	**M11^a^**	**M12^b^**	**M21^a^**	**M22^b^**	**M31^a^**	**M32^b^**
	**β** **(*****P*****)**	**β** **(*****P*****)**	**β** **(*****P*****)**	**β** **(*****P*****)**	**β** **(*****P*****)**	**β** **(*****P*****)**
Gender	0.030 (0.538)	−0.043 (0.381)	−0.077 (0.121)	−0.085 (0.078)	−0.052 (0.295)	−0.092 (0.055)
Age	**0.139** (0.014)	0.098 (0.080)	−0.012 (0.834)	−0.022 (0.683)	0.051 (0.365)	0.025 (0.649)
Education	−0.039 (0.348)	−0.031 (0.443)	−0.036 (0.392)	−0.055 (0.173)	−0.047 (0.260)	−0.059 (0.133)
Working experience	**−0.153** (0.006)	**−0.114** (0.036)	0.013 (0.819)	0.048 (0.365)	−0.057 (0.308)	−0.100 (0.849)
Department general practitioner						
Internist/pediatrician	0.052 (0.234)	0.055 (0.198)	−0.014 (0.742)	−0.006 (0.881)	0.011 (0.808)	0.019 (0.651)
Surgeon	0.031 (0.464)	0.003 (0.950)	0.084 (0.051)	**0.085** (0.040)	0.083 (0.055)	0.071 (0.082)
Gynecologist	−0.041 (0.410)	0.016 (0.739)	0.055 (0.272)	0.092 (0.055)	0.028 (0.571)	0.084 (0.074)
Chinese medical practitioner	0.024 (0.569)	0.008 (0.846)	−0.021 (0.608)	−0.002 (0.953)	−0.008 (0.856)	0.001 (0.973)
Work values						
Intrinsic values		**−0.080** (0.040)		**−0.073** (0.045)		**−0.098** (0.010)
Extrinsic values		−0.073 (0.061)		0.036 (0.348)		0.001 (0.961)
Reward values		**0.230** (<0.001)		**0.112** (0.004)		**0.194** (<0.001)
Social values		0.033 (0.397)		**0.280** (<0.001)		**0.248** (<0.001)
Altruistic values		**0.120** (0.002)		**0.150** (<0.001)		**0.180** (<0.001)
R^2^	0.007	0.076	0.003	0.117	0.001	0.135

*Boldface indicates statistical significance (P < 0.05);*

a *models M11, M21, M31 with controlled variables;*

b *models M21, M22, M32 with both controlled variables and work value dimensions*.

In summary, we found that primary physicians showed intentions to reduce antibiotic use, and the influence of work values on behavioral intentions of primary physicians were significant. Primary physicians intended to prescribe fewer antibiotics when perceiving higher intrinsic values, but they showed higher intentions to prescribe antibiotics when perceiving higher social values, more pursuit of reward values and greater emphasis on altruistic values.

## Discussion

The increasing frequency of antibiotic resistance has become a major health crisis. To our knowledge, this is the first study focused on the influence of work values on behavioral intentions regarding antibiotic use. Our study highlighted the intention of primary physicians to reduce antibiotic use, which was influenced by their work values. In this context, the existing literature provides similar results.

### Primary Physicians' Intentions to Reduce Antibiotic Use

Our results found that primary physicians showed intentions to reduce antibiotic use. Scholars have identified some interventions that can reduce prescribing behaviors or intentions among primary physicians (39–42). For example, a study showed that persuasive communication intervention could help to reduce physicians' intentions to inappropriate antibiotic use (β = 0.90, 95%CI: 0.41–1.38). General practitioners who received the persuasive communication intervention had a stronger behavioral intention to manage upper respiratory tract infection (URTI) without prescribing an antibiotic (Mean = 5.25, SD = 1.59) than those who did not receive it (Mean = 4.83, SD = 1.73), and they prescribed 0.47 fewer patients (39). Perz et al. found an 11% decrease in the rate of antibiotic prescribing among children by a multi-faceted educational intervention (95%CI: 8–14%, *P* < 0.001) (41, 42). A national trial in England found that the rate of antibiotic items dispensed per 1,000 population in the social norm feedback intervention group was 4.27 lower than that in the control group (3.3%; 95% CI = 0.957–0.977, *P* < 0.001), representing an estimated 73,406 fewer antibiotic items dispensed (42). Overall, these physicians' intentions to improve antibiotic prescribing behaviors is consistent with our study, indicating that the inappropriate use of antibiotics could be improved by more effective interventions.

### Primary Physicians' Work Values Influencing Behavioral Intentions

#### Primary Physicians Showed Intentions to Prescribe Fewer Antibiotics When Perceiving Higher Intrinsic Values

Our study indicated that primary physicians showed a stronger intention to reduce antibiotic prescriptions when perceiving higher intrinsic values. Previous studies also found the influence of intrinsic values on behavioral intentions (43, 44). A study showed a significant influence of work values on personal intention to leave (β = 0.289, *P* < 0.01), which was mediated by organizational supplies, because the individuals were more satisfied, experienced fewer psychological complaints, and had less intention to leave when organizations provided them with adequate intrinsic rewards (β = −0.790, *P* < 0.01) (43). Regarding to health workers, although scholars demonstrated that nurses in different sectors had various views on intrinsic values, they still considered it necessary to pay attention to intrinsic values (44). Moreover, some scholars indicated workers would have emotional exhaustion and burnout without adequate intrinsic rewards, which might eventually lead workers to behave contrary to desired goals (45), such as unwillingness to reduce antibiotic use. Therefore, improving primary physicians' intrinsic values may contribute to the reduction of antibiotic use.

#### Primary Physicians Showed Higher Intentions to Prescribe More Antibiotics When Perceiving Higher Reward Values, Social Values, and Altruistic Values

Firstly, primary physicians intended to prescribe more antibiotics if they strongly perceived the influence of social values. It was consistent with the notion put forward by psychologists that pursuit of social values was a basic human motivation (46). A previous study in the lifeguards found that perceived social values was a significant predictor of helping behavior (β = 0.65, *P* < 0.05), in which workers perceived their work as having a stronger duty and relationship with other people through heightened perceptions of social values, and resulted in investing additional time and energy in work (47). Other researches in medical field have also indicated that the enhancement of social recognition and the increase of social pressure could influence physicians' behavioral intentions, such as perceived higher patient pressure was associated with higher use of antibiotics (β = 0.102, *P* = 0.022) (11, 32). Thus, the attention to social values may broaden existing knowledge about the antibiotic prescribing behavioral intentions.

Secondly, primary physicians' intentions to prescribe antibiotics might increase if they put more emphasis on reward values (career promotion and self-enhancement). A longitudinal study of MBA graduates demonstrated that for both men and women, receiving rewards (such as promotion) made them intend to work longer per week (β = 0.108, P < 0.05; β = 0.170, *P* < 0.05) (48). Similarly, the emphases on rewards were also found in nurse group, with a study providing evidence that nurses perceived their skills and experience as extremely or quite unrewarded within the workplace (36% aged care, 42% public and 42% private), which might influence their work satisfaction thus affect their care decisions (44). Moreover, researches in the field of economics showed the influence of self-enhancement (authority and achievement) on entrepreneurial intentions (β = 0.147, *P* < 0.001), which also coincided with our results (26).

Thirdly, the positive influence of altruistic value on prescribing behavioral intentions indicated that primary physicians' emphasis on altruism made them more likely to prescribe antibiotics. The positive relationship between altruistic values and behaviors was confirmed in psychological researches (47, 49). A meta-analysis demonstrated perceptions that one could act to benefit others signified judgments of expectancy (effort will lead to effective performance, z = 6.02, *P* < 0.01) and instrumentality (effective performance will benefit others, z = 11.70, *P* < 0.01), motivating workers to invest additional time and energy in their work to achieve these outcomes (49). This view was confirmed by a study on the significance of task, in which the authors found that perceiving more altruistic values was able to enhance lifeguards' work risk spirit (β = 0.42, *P* < 0.05) (47). It also coincided with our results where primary physicians perceived their prescribing behaviors as altruistic, leading them to intend to prescribe more antibiotics. However, relevant literature pointed out that such altruistic behavioral intentions of physicians was likely to be subjective and might lead to inappropriate antibiotic use as well as impair the patient's health due to insufficient knowledge and complacency (11).

Overall, the primary physicians have shown a behavioral intention to change the inappropriate use of antibiotics. The relationship between behavioral intention and work values of primary physicians is complex. These results present evidences, which may contribute to more effective interventions for improving antibiotic prescribing in primary care facilities.

### Strengths and Limitations

This study is the first to link work values with specific antibiotic prescribing behavioral intentions, providing a new perspective for understanding antibiotic use and its management intervention in primary physicians. However, several limitations in this study are acknowledged. First, this study was conducted in primary care facilities in developing regions; but the generalization of the findings to other regions should be cautious. Second, in this study, behavioral intention was used as proxy measures for physicians' actual behavior. While there is evidence to suggest that behavioral intention is a reliable proxy for actual behavior, the deviation between actual behavior and intention still cannot be completely eliminated.

## Conclusion

The primary physicians have an intention to change the inappropriate antibiotic use. What's more, the influence of work values on the prescribing behavioral intentions regarding antibiotic use is significant. It suggests that training and education of work values may be an entry point for intervention when trying to improve public health and antibiotic stewardship efforts.

## Data Availability Statement

The datasets presented in this article are not readily available because the data that support the findings of this study are available from surveyed local institutions and governments but restrictions apply to the availability of these data, which were used under license for the current study, and so are not publicly available. Data are however available from the authors upon reasonable request and with permission of surveyed local institutions and governments. Requests to access the datasets should be directed to junyu_lu12@hust.edu.cn.

## Ethics Statement

The studies involving human participants were reviewed and approved by Ethics Committee of Tongji Medical College, Huazhong University of Science and Technology (NO: IORG 0003571). The patients/participants provided their written informed consent to participate in this study.

## Author Contributions

XZ conceived and designed the study. JL contributed to analysis, interpretation of data, and participated in writing of the manuscript. CL and DW participated in the cleaning and interpretation of data. All authors contributed to the article and approved the submitted version.

## Funding

This study was funded by the National Natural Science Foundation of China (grant no. 71373092).

## Conflict of Interest

The authors declare that the research was conducted in the absence of any commercial or financial relationships that could be construed as a potential conflict of interest.

## Publisher's Note

All claims expressed in this article are solely those of the authors and do not necessarily represent those of their affiliated organizations, or those of the publisher, the editors and the reviewers. Any product that may be evaluated in this article, or claim that may be made by its manufacturer, is not guaranteed or endorsed by the publisher.
